# Cytokinin and Ethylene Cell Signaling Pathways from Prokaryotes to Eukaryotes

**DOI:** 10.3390/cells9112526

**Published:** 2020-11-23

**Authors:** Baptiste Bidon, Samar Kabbara, Vincent Courdavault, Gaëlle Glévarec, Audrey Oudin, François Héricourt, Sabine Carpin, Lukáš Spíchal, Brad M. Binder, J. Mark Cock, Nicolas Papon

**Affiliations:** 1 Groupe d’Etude des Interactions Hôte-Pathogène, GEIHP, EA3142, UNIV Angers, SFR 4208 ICAT, F-49933 Angers, France; baptiste.bidon@univ-angers.fr (B.B.); samarkabbara@hotmail.com (S.K.); 2 Biomolécules et Biotechnologies Végétales, BBV, EA2106, Université de Tours, F-37200 Tours, France; vincent.courdavault@univ-tours.fr (V.C.); gaelle.glevarec@univ-tours.fr (G.G.); audrey.oudin@univ-tours.fr (A.O.); 3 LBLGC, University of Orléans, EA1207, INRA, USC1328, F-45000 Orléans, France; francois.hericourt@univ-orleans.fr (F.H.); sabine.carpin@univ-orleans.fr (S.C.); 4 Centre of the Region Haná for Biotechnological and Agricultural Research, Department of Chemical Biology and Genetics, Faculty of Science, Palacký University Olomouc, Šlechtitelů 27, CZ-783 71 Olomouc, Czech Republic; lukas.spichal@upol.cz; 5 Biochemistry & Cellular and Molecular Biology, University of Tennessee, Knoxville, TN 37996, USA; bbinder@utk.edu; 6 Algal Genetics Group, UMR 8227, Integrative Biology of Marine Models, Station Biologique de Roscoff, Sorbonne Université, UPMC, CNRS, F-29688 Roscoff, France; cock@sb-roscoff.fr

**Keywords:** ethylene, cytokinins, histidine kinases, receptors, cell signaling

## Abstract

Cytokinins (CKs) and ethylene (ET) are among the most ancient organic chemicals on Earth. A wide range of organisms including plants, algae, fungi, amoebae, and bacteria use these substances as signaling molecules to regulate cellular processes. Because of their ancestral origin and ubiquitous occurrence, CKs and ET are also considered to be ideal molecules for inter-kingdom communication. Their signal transduction pathways were first historically deciphered in plants and are related to the two-component systems, using histidine kinases as primary sensors. Paradoxically, although CKs and ET serve as signaling molecules in different kingdoms, it has been supposed for a long time that the canonical CK and ET signaling pathways are restricted to terrestrial plants. These considerations have now been called into question following the identification over recent years of genes encoding CK and ET receptor homologs in many other lineages within the tree of life. These advances shed new light on the dissemination and evolution of these hormones as both intra- and inter-specific communication molecules in prokaryotic and eukaryotic organisms.

## 1. CK and ET Signaling in Plants: Beyond the *Arabidopsis* Paradigm

Historically, cytokinins (CKs) and ethylene (ET) were primarily known as two prominent types of plant hormone (i.e., phytohormones) that regulate many aspects of plant development and physiology [1,2]. CKs share a common structure of *N*^6^-substituted adenine, with biological activities defined by the *N*^6^-substituent of isoprenoid or aromatic origin. They have pleiotropic functions. For instance, they were originally described as the major hormones regulating cell division but are also implicated in the control of morphogenesis and embryogenesis and inhibition of senescence. Conversely, ET is a simple gas, often referred to as the senescence hormone in plants, acting to stimulate senescence of leaves and petals as well as the ripening of fruits. Both CK and ET are also well known to orchestrate plant responses to many types of biotic and abiotic stresses [1,2].

**Figure 1:**
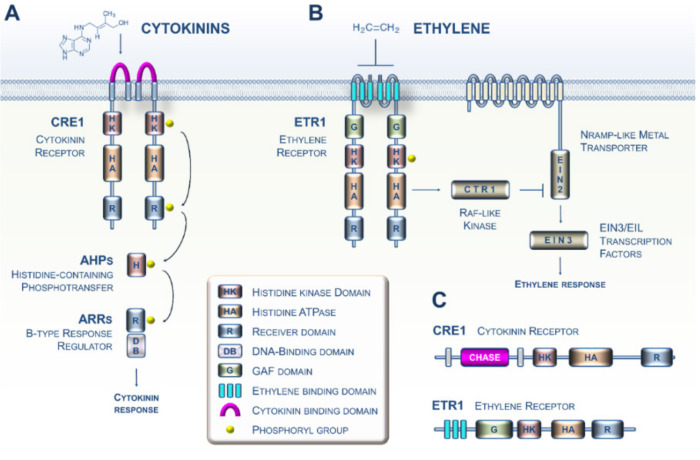
Perception and transduction of cytokinin (CK) and ethylene (ET) signals in the model plant *Arabidopsis*. (**A**) The cytokinin signaling pathway. The perception of CKs in *Arabidopsis* primarily involves the perception of these hormones by dimerized receptors such as the CRE1 receptor via the cyclase/histidine kinase-associated sensing extracellular (CHASE) domain. CRE1 then auto-phosphorylates (histidine kinase (HK) activity) and immediately transfers its phosphate group to the conserved histidine of a protein belonging to the histidine-containing phosphotransfer (HPt) family. This small protein then acts as a cytoplasm-to-nucleus shuttle and in turn phosphorylates a type B response regulator, which, when activated, positively regulates the transcription of response genes to the CK signal. (**B**) The ET signaling pathway. Ethylene molecules are detected by ethylene receptors (epitomized here by ETR1) with ethylene binding to the three transmembrane helices (in sky blue). Binding of ET to the dimerized ETR1 receptor downregulates its activity. In the absence of ET, ETR1 activates the serine/threonine kinase CTR1. The CTR1 protein then phosphorylates the EIN2 protein located in the ER membrane, leading to the proteolysis of EIN2. In the presence of ET, ETR1 activity is reduced, leading to less CTR1 activity; this leads to lower phosphorylation and accumulation of EIN2 protein and subsequent activation of the EIN3 and related transcription factors. EIN3 then positively regulates the transcription of ET signal response genes. (**C**) The domain structure of the *Arabidopsis* ET (ETR1) and CK (CRE1) receptors.

Because of their primary importance in plants, many investigations initiated in the 1980s aimed at identifying the sensing and transduction pathways of CK and ET in the model plant *Arabidopsis*. During the 2000s, these studies led to increasingly complex models with details about the mechanistic events governing CK and ET signaling (Figure 1A,B) [3,4]. Both phytohormone sensing circuitries in plants are related to the two-component systems typically described in prokaryotes [5,6]. More specifically, it is now well established that CKs and ET are perceived by two types of membrane-bound histidine kinase receptors, CRE1 and ETR1, respectively (Figure 1C) [7,8]. Importantly, CKs are perceived by the cyclase/histidine kinase-associated sensing extracellular (CHASE) domain of CRE1 (in pink, Figure 1A), whereas ET interacts with the ethylene-binding domain (ETBD) of ETR1, which consists of three transmembrane helices (in sky blue, Figure 1B) [9,10,11]. Mechanistically, the two pathways use fundamentally different families of downstream modules [5]. Based on a recent increase in Archaeplastida genomic resources, ranging from unicellular algae to land plants, there are now firm data showing that CK and ET sensing emerged in the green lineage, together with the corresponding biosynthetic pathways, during the terrestrialization process [12,13,14,15,16].

Although it has been known for many years that a wide array of non-plant organisms are able to synthesize and perceive both hormones, it has been supposed that canonical CK and ET signaling pathways are restricted to terrestrial plants [17,18]. In this viewpoint article, we first provide a summary of recent advances that have overturned this idea by showing that these signaling pathways are probably present in highly diversified prokaryotic and eukaryotic lineages. We then discuss the main evolutionary lines that have probably led to the currently observed distribution of CK and ET receptors within the tree of life.

## 2. CK and ET Signaling in Bacteria

**Figure 2:**
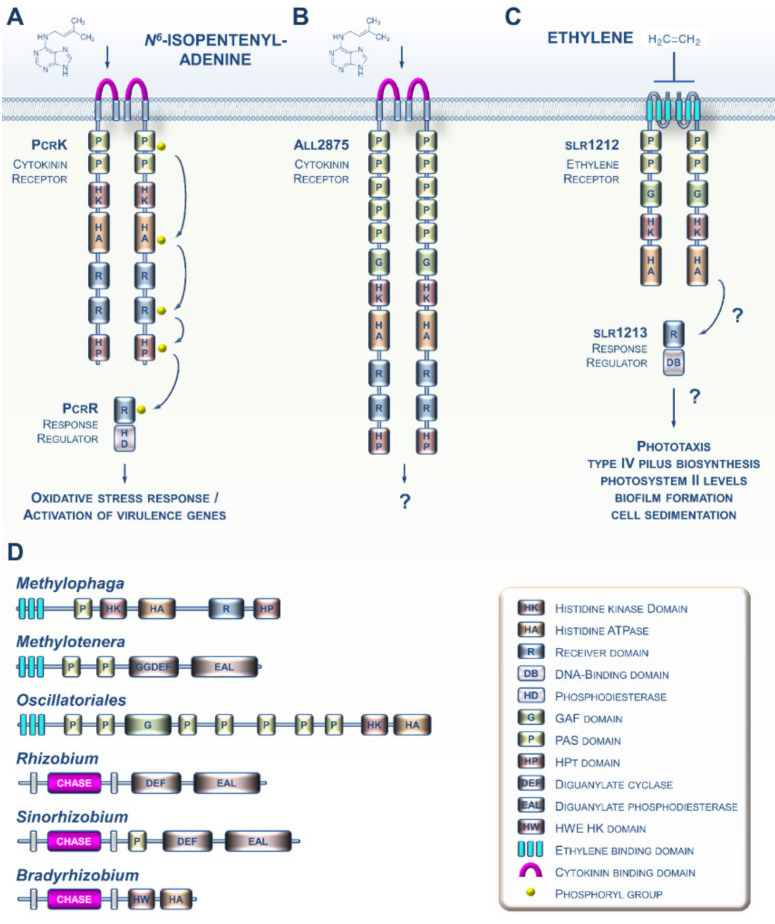
Current knowledge concerning CKs and ET signaling in bacteria. (**A**) In the phytopathogenic bacterium *Xanthomonas campestris*, PcrK is a CHASE-domain-containing HK receptor that binds the plant-produced CK *N*^6^-isopentenyladenine (iP). iP perception decreases PcrK HK activity and concomitantly the phosphorylation level of PcrR, the cognate RR of PcrK, to promote the phosphodiesterase activity of PcrR in degrading the second messenger (3′,5′-cyclic di-guanylic acid). This four-step phosphorelay signaling chain improves bacterial tolerance to oxidative stress by orchestrating the expression of a series of virulence-associated genes. (**B**) In the cyanobacterium *Nostoc* sp., all2875 is a CHASE-domain-containing HK receptor that moderately binds iP and, with lower affinity, *trans*-zeatin. (**C**) ET signaling in the cyanobacterium *Synechocystis* sp. ET negatively regulates the ETR-like protein slr1212, which putatively signals to a downstream response regulator protein, slr1213. The GAF domain binds a chromophore and functions as a light receptor, making this a bifunctional receptor. (**D**) Some other examples of domain arrangement for CK and ET receptor homologs found in various bacteria.

Various prokaryotes are capable of producing both CK and ET [19,20]. Although the biosynthesis of both hormones as cell-to-cell communication molecules in prokaryotes is not a new observation, the discovery of sensing circuitries for these molecules in prokaryotes is more recent. For instance, it was shown a few years ago that the Gram-negative bacterium *Xanthomonas campestris*, which causes black rot disease in crucifers, is capable of perceiving the cytokinin *N*^6^-isopentenyladenine (iP) from the host plant using the PcrK histidine kinase receptor (Figure 2A). Sensing of plant-derived CKs by the bacterium leads to the expression of many genes, including those involved in resistance to oxidative stress, thus enhancing bacterial resistance to the host’s defenses [21,22]. Another study recently provided evidence that the cyanobacterium *Nostoc* encodes a CK receptor homolog (Figure 2B), although it has yet to be demonstrated to be a functional receptor [23]. Most of the advances concerning ET signaling in prokaryotes were gained from studies of the cyanobacterium *Synechocystis* (Figure 2C) [20,24,25]. Although the occurrence of putative ET receptors in cyanobacteria has been known for over 20 years [26], it was only recently that data emerged suggesting that the *slr1212* gene encodes a bona fide ET receptor governing various processes underlying cell motility [20].

An increasing amount of genomic data suggests that CK and ET receptors have evolved and diversified throughout the prokaryotic domain. For instance, potential CK receptor homologs with CHASE domains are found in various plant pathogenic and symbiotic bacteria (Figure 2D) [27]. Similarly, many species of proteobacteria and cyanobacteria possess genes that are predicted to encode proteins with an ETBD [28]. These proteins show a wide range of putative output domains, from simple to complex, and even domains not found in plant receptors (Figure 2D). Altogether, this shows us that these prokaryotic CK and ET receptor homologs are likely to have diverse biochemical outputs and may also integrate multiple input signals in addition to CKs and ET.

## 3. CK and ET Signaling in Opisthokonta

**Figure 3:**
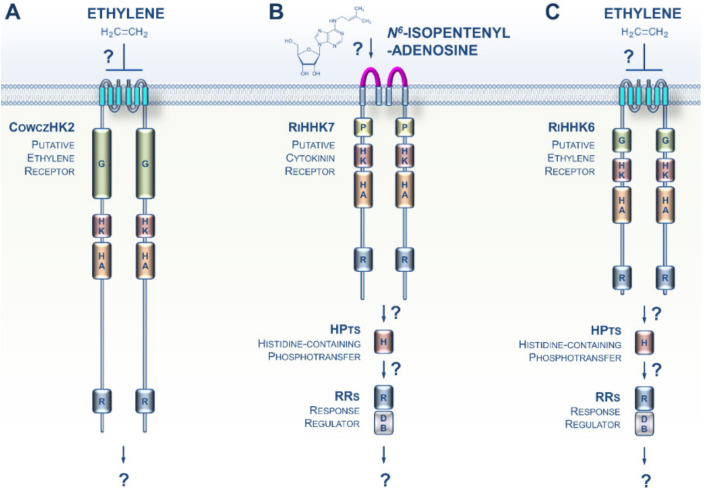
CK and ET signaling pathways potentially present in Opisthokonta. (**A**) The Mesomycetozoa *Capsaspora owczarzaki* is a representative of the closest known unicellular clade to animals. A recent phylogenomic analysis identified an ET receptor homolog in this species, CowczHK2. (**B**) A putative CK receptor was recently identified in the clade of Glomeromycotina. (**C**) Glomeromycota genomes also encode homologs of plant ET receptors, but their functions remain undefined. For a domain key, please refer to Figure 2.

The Opisthokonta lineage includes animals (Metazoa), fungi, choanoflagellates, and Mesomycetozoa, and therefore represents a supergroup of morphologically highly diversified species [28]. A recent phylogenomic analysis identified a putative ET receptor in *Capsaspora owczarzaki*, a representative in the closest known unicellular clade relative of animals, i.e., the Mesomycetozoa (Figure 3A) [29]. Thus, an ET signaling pathway was possibly present in the ancestors of animals where it would have regulated unknown cellular processes. However, if true, this feature was lost early during the evolution of the multicellular animal lineage.

Whereas typical histidine kinase proteins are lacking in Metazoa, it has been known for more than two decades that this family of prominent sensing proteins expanded in the fungal kingdom with a distribution related to their different lifestyles [30,31]. However, although it was previously established that both fungal- and plant-produced CKs and ET play crucial roles in orchestrating some features of the various modes of fungi–plant interaction (symbiosis or pathogenicity), how fungi sense CKs and ET has remained elusive [32,33,34,35,36,37,38]. Recent genomic resources from early-diverging fungi (EDF) revealed that plant-like CK and ET receptors appeared early in the evolution of fungi [39]. This trait is particularly noticeable in the EDF that closely interact with roots or decaying plant material. Supporting this idea, CK and ET receptor homologs were recently identified in the clade Glomeromycotina, which includes obligate symbionts that colonize more than half of the plant population on earth (Figure 3B,C) [40]. The presence of these receptor homologs, coupled with prior data showing a pivotal role of plant-derived CKs and ET in various Glomeromycota–plant interactions, as well as recent descriptions of CK and ET biosynthesis in EDF, [41] strongly suggest the hypothesis that early in evolution, even before the terrestrialization process, plants and fungi developed and retained closely related CK and ET receptors that are likely to be important for cross-kingdom signaling. Therefore, CK- and ET-mediated communications have probably played an essential role in land colonization by plants and fungi.

## 4. CKs and ET Signaling in Amoebozoa

Although they sometimes develop as social organisms, most Amoebozoa are unicellular and are described as behaving either as free-living cells in water and soils or as parasites of humans and other eukaryotes [28]. This may explain why the histidine kinase sensor family has expanded in these protists to dynamically sense environmental cues [29].

**Figure 4:**
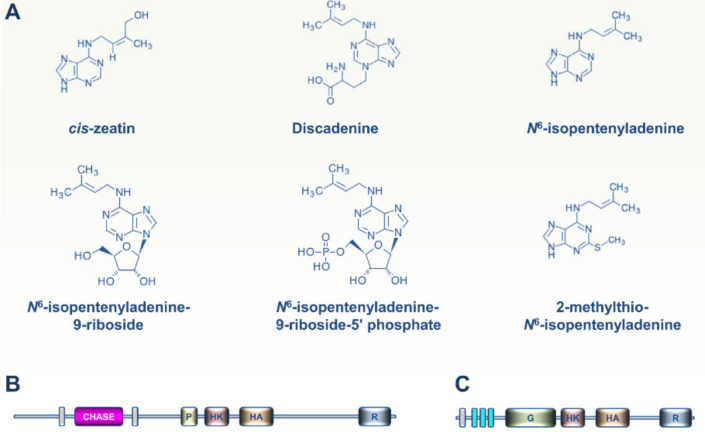
CKs produced in slime molds and CK and ET signaling modules in Amoebozoa. (**A**) Six different CKs were identified recently in the slime mold *D. discoideum*. CKs were previously shown to coordinately orchestrate the different developmental stages of this social amoeba, especially spore formation. (**B**) The *D. discoideum* DhkA histidine kinase sensor contains a CHASE domain and was also shown to play a role in the CK-controlled sporulation process. To date, genetic evidence for a role for DhkA in transmitting the CK signal is still lacking. (**C**) Recent phylogenomic analysis revealed the presence of ET receptor homologs in free-living amoebae (*Acanthamoeba* and *Balamuthia* sp.), but not in other Amoebozoa clades.

Little information is available concerning the occurrence of CKs and ET signaling pathways in Amoebozoa. Six different CKs were recently identified in the slime mold *Dictyostelium discoideum* (Figure 4A) [42]. CKs were previously shown to coordinately orchestrate the different developmental stages of this social amoeba, especially spore formation [43,44,45]. In this regard, the *D. discoideum* DhkA histidine kinase sensor contains a CHASE domain and was also shown to play a role in sporulation (Figure 4B) [46]. To date, there is no evidence that DhkA has a role in transmitting the CK signal.

More recent phylogenomic analysis revealed the presence of putative ET receptors in free-living amoebae (notably *Acanthamoeba* and *Balamuthia* sp.) (Figure 4C) but not in other Amoebozoa clades [29]. As hypothesized above for Mesomycetozoa, it is likely that ancestral amoebae developed ET receptors and sensing circuitries for cell-to-cell communication, but lost this transduction system during the evolutionary paths to extant Amoebozoa [17].

**Figure 5:**
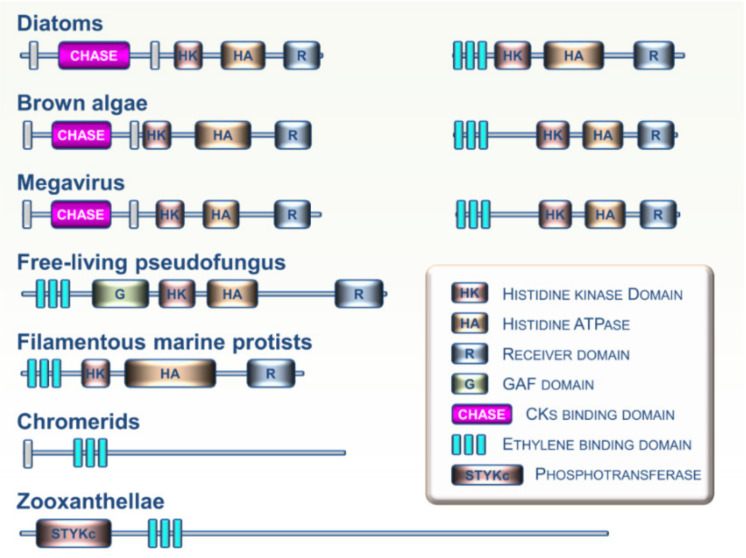
Examples of domain arrangements for CK and ET receptor homologs found in various members of the Stramenopiles, Alveolates, and Rhizaria (SAR) supergroup.

The Stramenopiles, Alveolates, and Rhizaria (SAR) supergroup includes highly diversified unicellular and multicellular organisms ranging from algal and planktonic species to ciliates and oomycetes [28]. Analysis of growing SAR genomic resources indicates that CK and ET receptors homologs are not widespread in this lineage. However, some CK and ET receptor homologs were recently identified in several SAR clades including zooxanthellae, chromerids, free-living pseudofungus, filamentous marine protists, diatoms, and brown algae (Figure 5) [17,29]. Analysis of domain arrangements from these putative CK and ET sensing proteins suggests that these receptors may have a broad range of biochemical outputs. To date, no functional studies have been performed on these candidate proteins to determine if they are involved in the perception of CKs or ET. However, it cannot be ruled out that future research on these particular organisms will show a major role for these hormones and their signaling pathways in the physiological processes closely linked to their specific lifestyles in various aquatic environments. Based on genome analysis, evidence indicates possible exchanges of histidine kinase genes between brown algae and giant DNA viruses [47]. Such exchanges suggest a possible role for viruses as mediators of horizontal transfers of CK and ET receptors between eukaryotic lineages. This may be an important factor in understanding phylogenetic relationships between genes and their distribution across the different branches of the tree of life.

## 5. Viewpoint: An Evolutionary Perspective of CKs and ET Perception within the Tree of Life

Given the diversity of organisms that contain putative CK and ET receptors, it is time to update the viewpoint that CKs and ET are solely hormones that affect plants. At present, only a small community of specialists on these hormones considers a much broader role for CKs and ET in the physiology of non-plant organisms and their research shows that these molecules are among the oldest and most widespread intra- and interspecific communication molecules [17,18]. It is likely that both classes of molecule arose during Earth’s early history, since CKs could have appeared first as modified nucleic acid byproducts and since ET is produced by many microbes and is abiotically generated from interactions of light with dissolved organics [19,48,49]. Biosynthesis of CKs and ET occurs in diverse prokaryotic and eukaryotic lineages [11,20,41,42,50,51,52,53,54]. In addition, ever-expanding genomic resources indicate that homologs of plant CK and ET receptors occur widely in basal lineages of the tree of life [17,29,39]. Taken together, all these elements support the idea that CKs and ET were probably broadly present on the ancient Earth and that these simple compounds were co-opted as signaling molecules by early organisms.

Given this, future research needs to explore the roles of these receptors and delineate signaling pathways in more non-plant species. These analyses should include tests of whether the putative receptors bind CKs or ET. If they do, it will be important to investigate the physiological roles of binding in the organism [55,56]. In many cases, it will also be relevant to determine the biochemical outputs and subcellular locations of the receptors to fully understand their functions in the cell [57,58]. Finally, it will be necessary to continue to refine the phylogenetic analyses of these receptors to understand how they arose and evolved in prokaryotes and non-plant eukaryotes.

Another important perspective in the field thus concerns the search for mechanisms of phytohormone perception in organisms that use these signals in their interactions with plants, in both symbiotic and pathogenic contexts. For instance, although it has been known for many years that insects are able to manipulate plant CKs, it has only very recently been proposed that arthropods also synthesize CKs [52,59,60]. Similarly, there is increasing evidence of roles for fungal CKs in promoting the virulence of plant pathogenic molds (filamentous Ascomycota) [34,37]. A final and important example in this respect is the ability of plant-parasitic nematodes to synthesize CK derivatives to manipulate the host system and establish long-term parasitic interactions [61,62]. These recent studies strongly justify the search for molecular perception mechanisms in these models. Finally, the studies highlighted above show the importance of conducting similar investigations on other phytohormones, such as brassinosteroids, auxins, strigolactones, jasmonates, salicylic acid, abscisic acid, and gibberellins, given their roles in various types of plant biotic interactions [63,64].

The next decade of active research in this field will shed new light on the dissemination and evolution of phytohormones as both intra- and inter-specific communication molecules in prokaryotic and eukaryotic organisms.
